# Human Activity Recognition Using Hybrid Coronavirus Disease Optimization Algorithm for Internet of Medical Things

**DOI:** 10.3390/s23135862

**Published:** 2023-06-24

**Authors:** Asmaa M. Khalid, Doaa Sami Khafaga, Eman Abdullah Aldakheel, Khalid M. Hosny

**Affiliations:** 1 Information Technology Department, Faculty of Computers & Informatics, Zagazig University, Zagazig 44519, Egypt; asmaa.elhenawy@gmail.com (A.M.K.); k_hosny@yahoo.com (K.M.H.); 2 Department of Computer Sciences, College of Computer and Information Sciences, Princess Nourah bint Abdulrahman University, P.O. Box 84428, Riyadh 11671, Saudi Arabia; dskhafga@pnu.edu.sa

**Keywords:** feature selection, population, human activity recognition, optimization, smartphones, sensors

## Abstract

Background: In our current digital world, smartphones are no longer limited to communication but are used in various real-world applications. In the healthcare industry, smartphones have sensors that can record data about our daily activities. Such data can be used for many healthcare purposes, such as elderly healthcare services, early disease diagnoses, and archiving patient data for further use. However, the data collected from the various sensors involve high dimensional features, which are not equally helpful in human activity recognition (HAR). Methods: This paper proposes an algorithm for selecting the most relevant subset of features that will contribute efficiently to the HAR process. The proposed method is based on a hybrid version of the recent Coronavirus Disease Optimization Algorithm (COVIDOA) with Simulated Annealing (SA). SA algorithm is merged with COVIDOA to improve its performance and help escape the local optima problem. Results: The UCI-HAR dataset from the UCI machine learning repository assesses the proposed algorithm’s performance. A comparison is conducted with seven well-known feature selection algorithms, including the Arithmetic Optimization Algorithm (AOA), Gray Wolf Optimizer (GWO), Whale Optimization Algorithm (WOA), Reptile Search Algorithm (RSA), Zebra Optimization Algorithm (ZOA), Gradient-Based Optimizer (GBO), Seagull Optimization Algorithm (SOA), and Coyote Optimization Algorithm (COA) regarding fitness, STD, accuracy, size of selected subset, and processing time. Conclusions: The results proved that the proposed approach outperforms state-of-the-art HAR techniques, achieving an average performance of 97.82% in accuracy and a reduction ratio in feature selection of 52.7%.

## 1. Introduction

The IoMT is the newest IoT age, rapidly attracting researchers’ interest due to its extensive use in SHS. IoMT involves integrating medical devices, hardware, and software applications through a network to provide more efficient and easier healthcare services to patients and to increase the consistency and precision of medical devices [1]. An IoMT-based intelligent healthcare system involves various phases: smart sensors are connected to the patient’s body through wearable devices to capture medical data; the gathered data are transmitted through the network to medical experts for analysis and prediction with the help of smart applications; and finally, feedback information from the physician can be returned to patients through smartphone application [2]. HAR is one of the emerging applications of IoMT, which plays a crucial role in healthcare systems. HAR is a process by which the detailed actions of a person’s daily life (walking, running, and standing) are recorded by smart sensors to recognize the correct activity [3]. Recognizing patients’ activities is crucial, particularly in elderly care, remote patient monitoring, and rehabilitation support.

In recent decades, various approaches have been employed for HAR, including computer vision [4], wireless signals [5], installed sensors [6], wearable sensors [7], and smartphone devices [8]. However, these techniques have limitations, such as the need for cameras and good light conditions in computer vision methods, restricted wireless signal monitoring areas, and users’ uncomfortable feelings when using wearable sensors [9]. Finally, smartphone devices are considered the most appropriate method to record human activities for many reasons, including wide availability, ease of use, and efficiency and safety when collecting data [10].

An FS problem can be formulated as an optimization problem in which the objective is to find the subset of data with the minimum size and maximum classification accuracy. Due to the enormous amount of data obtained from various sensors, an efficient FS approach is needed to select the most relevant data, reduce data dimensionality, maximize classification accuracy, and minimize computational complexity. For this reason, various metaheuristics have been applied to solve the problem, such as the ABC algorithm [11], WOA [12], AOA [13], MPA [14], HHO [15], FDO [16], CSO [17], SDBO [18] and many others [8,19].

Although most metaheuristics perform well in solving the FS problem, they have significant shortcomings, including being prone to local optima, having limited diversity, and increasing computational time [20]. For this reason, researchers attempted to apply the hybridization between two metaheuristics to benefit from their advantages and treat their limitations.

This paper proposes an efficient approach for selecting the most relevant and highest classification accuracy feature subset from the HAR dataset, which helps precisely identify human activities. The proposed approach also minimizes the selected subset’s size to reduce data dimensionality and computational cost. The proposed approach uses the hybrid COVIDOA with the simulated annealing (SA) algorithm. COVIDOA and SA are well-known metaheuristics that proved their effective performance in solving various optimization tasks [21,22]. However, we propose a hybrid approach to improve the solutions’ diversity and minimize the two method’s limitations.

The contributions of the paper are summarized as follows:

- This study aims to present an efficient HAR system to classify human activities accurately.
- The proposed system is based on hybridizing efficient COVIDOA with SA to produce an efficient feature selection model, and then using different well-known classifiers to classify the selected features.
- In the proposed model, we compared four classifiers to determine which classifier best suits the activity classification process.
- The KNN classifier proved its superiority in classification against other classifiers.
- Various metrics are used for evaluation, including classification accuracy, best fitness, selected subset size, and processing time.
- The proposed COVID-SA + KNN model is used for HAR of the public UCI-Human Activity Recognition (UCI-HAR) dataset and showed excellent classification results compared to various state-of-the-art techniques.

The remainder of this paper is organized as follows: Section 2 provides an overview of the related work. Section 3 describes the materials and methods of this work, including dataset description, COVIDOA, and SA algorithms. The proposed COVIDOA-SA algorithm is presented in Section 4. The evaluation metrics and numerical results are discussed in Section 5. Finally, the paper is concluded in Section 6.

## 2. Related Work

This section briefly overviews the recent studies proposed for sensor-based HAR applications using various public datasets. Kwon et al. [23] proposed unsupervised learning techniques for HAR using data collected from smartphone sensors. The results showed that the accuracy of the proposed approach is maximized for HAR without generating the training dataset by hand. Ronao and Cho [24] proposed an efficient HAR system using smartphone sensors. The proposed algorithm exploits the inherited characteristics of activities. The algorithm outperforms state-of-the-art HAR techniques, achieving an average performance of 94.79% in accuracy.

A transition-aware HAR (TAHAR) system is proposed to recognize smartphone physical activities [25]. The outcomes demonstrate that TAHAR is superior to the existing techniques and reveals the architecture’s main advantages. A hybrid FS approach is utilized for HAR in [26]. This model used a sequential floating forward search (SFFS) to select the most suitable features for activity recognition. The support vector machine was then used to classify the selected features. The proposed approach achieved efficient performance and superior classification results. Hassan et al. [27] proposed an approach for HAR from smartphone sensors by extracting features from raw data.

Comparing traditional approaches showed that the proposed approach has superior HAR performance. In [28], an accelerometer-based architecture is designed for HAR using smartphones. The smartphone extracts data about the participant’s daily activities in this approach. The data are then preprocessed, denoised, normalized, and segmented to obtain the most important feature vectors. The Deep Belief Network (DBN) is then used for training the features for a robust activity recognition system.

In addition, a Convolution Neural Network is proposed for the real-time classification of human activities. The results demonstrate that the proposed method exceeds the others based on two large public datasets: UCI-HAR [29] and Pamap2 [30]. Zhou et al. [31] built a deep-learning framework for an accurate HAR system. This framework develops an intelligent auto-labeling approach based on Deep Q-Network (DQN) with a distance-based reward rule, enhancing learning efficiency in IoT environments. In addition, a Long Short-Term Memory (LSTM)-based classification method is proposed to identify fine-grained patterns. The experiments demonstrate the proposed method’s efficiency using real-world data.

Much work has been undertaken regarding HAR using smartphone data for the last three years. For example, Sakorn and Anuchit [32] proposed a generic system for HAR in smart homes using smartphone data. The proposed system is based on four LSTM networks to study the recognition performance. The public UCI-HAR dataset [29] was considered for testing, and the results demonstrate that the proposed four-layer LSTM system outperforms the other LSTM networks with an accuracy of 99.39%. Luptáková et al. [33] proposed an alternative approach, called a transformer, for classifying human activities. The advantages of the proposed transformer model include directly focusing on using attention mechanisms to find correlations in the time series between features and the longer path length between features, allowing more accurate learning of the context in long time series.

Another novel HAR system is proposed in [13] by optimizing CNN and AOA. CNN is utilized to learn and extract the features from the dataset, and AOA is used to select the optimal features. The SVM classifier is used to classify the selected features. The proposed model achieved a classification accuracy of 95.23%, 99.5%, and 96.8% for UCI-HAR [29], WISDM-HAR [34], and KU-HAR [35] datasets, respectively.

Recently, Suh [36] proposed a novel transformer-based adversarial learning framework for HAR using TASKED. This model adopts the teacher-free self-knowledge distillation to improve HAR’s stability and performance. The results showed that the proposed model outperforms the previous techniques and effectively improves subject generalization. Zhang et al. [37] proposed a deep learning model, ConvTransformer, for HAR. The proposed used CNN to model the local information of the sensor signal, and then a transformer to obtain the correlation of the feature sequence, and an attention mechanism to highlight essential features. A comparison with state-of-the-art techniques showed that the proposed model is more robust and has higher classification accuracy. The existing recent studies are summarized in Table 1.

## 3. Materials and Methods

### 3.1. UCI-HAR Dataset

UCI-HAR is a public dataset published by Anguita et al. [41] for the daily activities of humans. Thirty participants aged 14 to 48 years old were required to apply the experiments using a smartphone on the waist. The activities are classified into six groups: WK, WU, WD, ST, SD, and LD. Three-axial linear acceleration and angular velocity were captured using the accelerometer and gyroscope with a sampling frequency of 50 Hz. Noise filters were used to preprocess the accelerometer and gyroscope signals before the samples were taken in fixed-width sliding windows of 2.56 s and 50% above. Body acceleration was then isolated from the gravity acceleration component to extract representative features properly. A fixed-width sliding window of 2.56 s was used to segment the signals with 50% overlapping.

### 3.2. COVIDOA

This section introduces the main stages of the novel COVIDOA. In general, COVIDOA simulates the replication mechanism of coronavirus particles [21]. The COVIDOA has two main parameters upon which its performance depends. The first is the number of proteins (NoP), representing the number of generated viral proteins during replication. In contrast, the second parameter is the mutation rate (MR), which represents the probability of applying mutation by the coronavirus proteins. The stages can be summarized as follows:

- Virus entry and uncoating

The virus releases its viral contents inside the human cell after entry through a structural protein called a spike.

- Replication using the frameshifting technique

Millions of viral proteins are then created using the frameshifting mechanism. The created proteins are then merged to form new virions. This stage can be modeled using the following equations:

(1) $$V_{i}(1)=rand\left(lb,ub\right),$$

(2) $$V_{i}\left(2:D\right)=P(1:D-1)$$

where *P* is the parent protein sequence, $V_{i}$ is the new child protein number *i*, and *lb*, ub, and *D* represent the lower bound, upper bound, and dimension of the problem.

- Mutation

The virus applied mutation to trick the human immune system as follows:

(3) $$Y_{i}=\left\{\begin{array}{c} r\;if\;rand\left(0,1\right)<MR\\ V_{i}\;otherwise \end{array}\right.$$

where *Y* and *V* are the old and mutated solutions. *i* = 1, …, *D*. and *r* is a random number that takes a value between lb and ub. *MR* represents the mutation rate, the value of which ranges from 0.005 to 0.5.

- New particle formation

**Figure 1:**
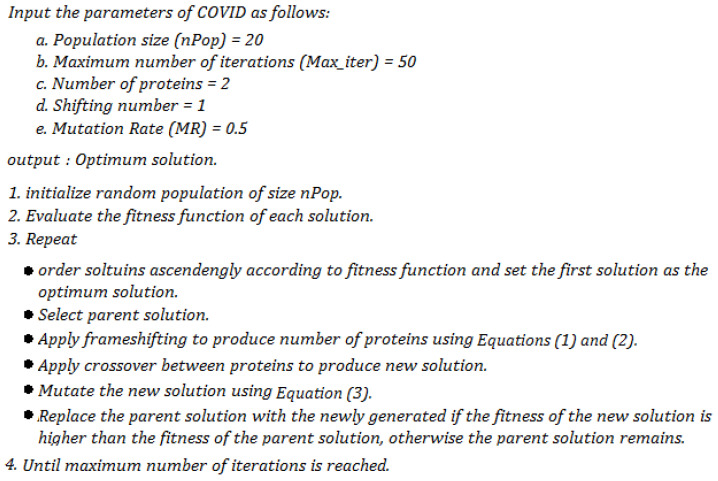
Pseudocode of COVIDOA.

The new proteins generated in the previous stage are grouped to form new particles, which will then be released from the current human cell targeting the new one. The pseudocode of COVIDOA is shown in Figure 1.

### 3.3. SA Algorithm

SA is a single-solution metaheuristic that mimics the annealing process of metals. Annealing is a process of making metals more workable by slowly cooling down their temperature. SA starts with an initial temperature of *T*_0_ and a randomly selected candidate solution.

SA gradually updates the initial random solution by selecting a neighboring solution. If the neighboring solution is better, the initial and optimum solutions are updated, which is repeated until reaching the final temperature. The initial temperature is the highest and is gradually decreased using a parameter called cooling rate *l* until it reaches the final lowest temperature Tend.

The temperature is updated using the following formula:

(4) t=t×l,0<l<1

where *t* is the current temperature and *l* is the cooling rate.

To avoid becoming trapped in a local optima problem, SA can accept a new, worse neighboring solution according to acceptance criteria of how much worse the new solution is and the current temperature according to the current formula:

(5) $$\exp\left(\frac{\omega }{t}\right)\leq rand$$

ω represents the difference between the current and new neighboring fitness, and *t* is the current temperature.

## 4. The Proposed Hybrid Approach

**Figure 2:**
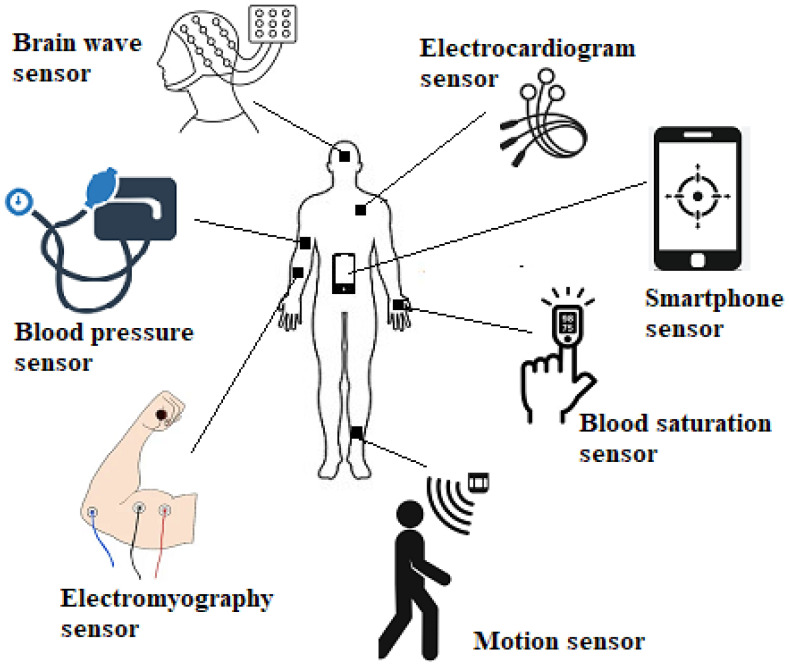
Common human body sensors.

Most IoMT applications include human body sensing, which allows them to collect data precisely and use the body’s vital indicators as their collecting objectives. Wearability is the most common requirement for gathering human body vital signs. The most commonly used human body sensors are shown in Figure 2.

**Figure 3:**
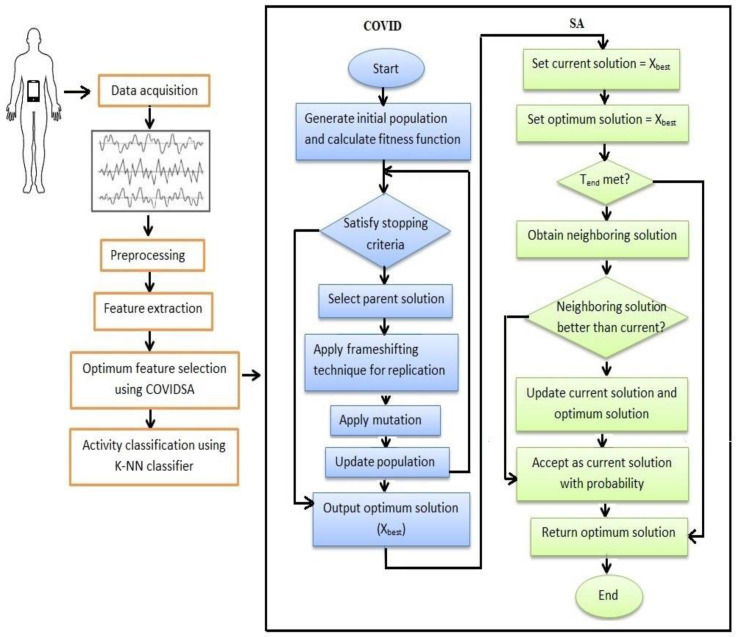
The proposed HAR model uses a hybrid COVID-SA algorithm.

This section illustrates the stages of the proposed HAR system based on the hybrid COVID-SA algorithm. The flowchart in Figure 3 shows the stages of the proposed approach. In the COVID-SA algorithm, COVID first works on the training and testing data of the UCI-HAR dataset to select the most relevant features. After COVID finishes its work, SA takes the optimum solution obtained by COVID as its initial solution instead of working with the initial random solution. SA is used to improve the performance of COVID as it can escape from the local optima trap. The stages of the proposed approach are as follows:

- Preprocess the dataset by splitting it into training and testing partitions.
- Determine the initial values for the COVID algorithm’s parameters, such as population size (*nPop*), maximum number of iterations (*Max_iter*), problem dimension (*D*), number of proteins (*NoP*), and mutation rate (*MR*).
- Generate initial binary population of solutions and compute fitness function. The initial binary population is generated by using the sigmoid function [36], which is one of the S-shaped transformation functions that transform the real-valued solution to its binary representation as follows [42]:

(6) $$S\left(X\right)=\frac{1}{1-e^{-X}},X_{B}=\left\{\begin{array}{c} 1\;if\;rand()\geq S(X)\\ 0\;otherwise \end{array}\right.$$

where $X_{B}$ is the binary form of solution X.

To calculate the fitness function of the initial population, the selected features (the feature corresponding to 1) are fed into the KNN classifier to calculate the fitness of the initial population value as follows:

(7) $$fitness=a\epsilon +(1-a)\frac{SSize}{TSize}$$

where a is a random value in the range [0, 1], ϵ is the error rate of the classifier, and SSize and TSize are the size of the selected subset of features and the total size of features, respectively.

- Select a parent solution for replication using roulette wheel selection and apply replication to produce several viral proteins using Equations (1) and (2), and then apply crossover between generated proteins to form a new virion (solution).
- Apply mutation to the solution generated in the last step to obtain the mutated solution using Equation (3).
- Update the population for the next generation.
- Repeat steps 4 to 6 until the termination criterion is met and get the optimum solution.
- Initialize the SA algorithm’s parameters, such as T0, Tend, and l, and set the optimum solution obtained from the COVID algorithm in the last step as the initial solution of SA.
- Select a new neighboring solution and update both the current solution and the best solution according to the current temperature and how worse the new solution is using Equation (5)
- Update the temperature according to the cooling rate l.
- Repeat steps 9 and 10 until the final temperature is reached.
- Evaluate the obtained optimum solution using various evaluation metrics such as accuracy, best fitness, average fitness, STD, and feature subset size, as discussed in the next sections.

## 5. Experimental Results and Discussions

### 5.1. Data Preprocessing

The samples were preprocessed with a median filter for noise removal before being added to the dataset. The total number of samples in the dataset is 10,299, separated into training and testing sets. Authors have published data files where 7352 (71.39%) samples represent the training set, and the remaining 2974 samples (28.61%) represent the testing set. The details of UCI-HAR activities can be found in [29].

### 5.2. Parameter Setting

To prove the superiority of the proposed approach, we conducted a comparison with seven well-known metaheuristics, including AOA [13], GWO [43], WOA [12], RSA [44], ZOA [45], GBO [46], SOA [47], and Coyote Optimization Algorithm (COA) [48]. For a fair comparison, we used a population size of 20 and a maximum number of iterations of 50 for the proposed and competing metaheuristics. The results may differ because of the use of random numbers in the optimization process. For this reason, we executed each algorithm 20 times and took the average results. All algorithms were executed on a DELL laptop with Intel (R) Core (TM) i7-1065G7 processor, 8.0 GB RAM, and Windows 10 Ultimate 64-bit operating system. MATLAB R2016a was used to develop and run all the algorithms. The parameters of the utilized state-of-the-art algorithms were set as provided in Table 2.

### 5.3. Evaluation Measures

Various metrics are used to prove the effectiveness of the proposed COVID-SA algorithm in FS, where the performance metrics are defined as follows:

(8) $$Accuracy\left(ACC\right)=\frac{TP}{TP+TN+FP+FN}$$

(9) $$Precision\left(Pre\right)=\frac{TP}{TP+FP}$$

(10) $$Recall/Sensitivity=\frac{TP}{TP+FN}$$

*TP*, *TN*, *FP*, *and FN* represent true positive, true negative, false positive, and false negative classification rates.

In addition to the previous metrics, additional evaluation measures are utilized, such as the best cost, the average cost (AVG), the corresponding STD, and processing time. These additional metrics are used to prove the ability of the utilized metaheuristics to obtain the optimum solution for the FS problem.

### 5.4. Parameter Tuning for Different Classifiers

Table 3 explains the parameters of KNN, DA, and DT classifiers. With the trial-and-error approach, which is widely used for parameter selection, it is found that the parameter values that achieve the best classification results for these classifiers are: K = 5 and the distance function is Euclidean for KNN classifier, max depth = 4, and the criterion is Gini for DT classifier, and Gamm = [0:0.1:1] and Delta = 0 for DA classifier. The NB has almost no hyperparameters to tune, so it usually generalizes well.

### 5.5. Numerical Results and Analysis

In the experiments, we employed four well-known classifiers for applying feature classification: KNN, DA, NB, and DT. A comparison is conducted between the four classifiers to determine the most fitting for the HAR problem. The numerical results obtained for the UCI-HAR dataset according to classification accuracy, best cost, average cost, STD, selection size, and execution time (in minutes) for all classifiers are shown in Table 4. It is obvious from the table that the KNN classifier has better classification results than the others in terms of accuracy, best cost, average cost, and selection size. It achieved the highest classification accuracy (0.9782) and the lowest best cost, average cost, and selection size values (0.02455, 0.0281, and 265). In terms of STD and processing time, the DA classifier is the best. However, KNN exceeds it in terms of the remaining measures.

One of the common evaluation techniques for different classification models is the confusion matrix. The confusion matrix shows how our classification model is confused when it makes predictions, where the confusion matrix involves two common error patterns as follows:

- False Positive: the model predicted positive, and it is false. For example, the model predicted that the activity is walking, but it is not (it is standing, for example).
- False Negative: the model predicted negative, and it is false. For example, the model predicted that the activity was not walking, but it was.

A detailed explanation and analysis of the confusion matrix obtained from the four mentioned classifiers are presented as follows:

A. K-Nearest Neighbor (KNN) classifier

**Figure 4:**
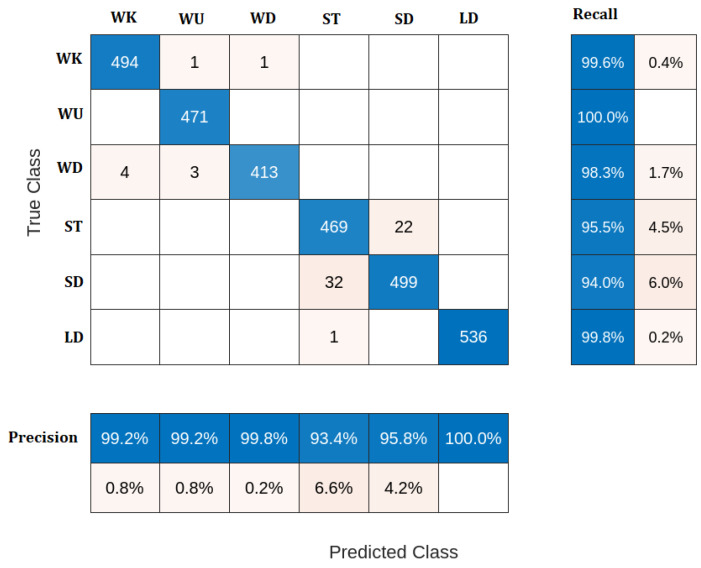
Confusion matrix of KNN classifier.

KNN is one of the simplest and most well-known classifiers. It works by comparing the similarity of a new sample with the other samples. The distances between the incoming and other samples are calculated using a predefined distance function. In this work, the Euclidean distance function is utilized. In KNN, the new sample is assigned to a class to which most of the closest K neighbors belong. In the proposed algorithm, k is set to 5. The KNN classifier’s confusion matric, precision, and recall results are shown in Figure 4. As seen from the figure, The total number of walking (WK) activity samples is 496. The KNN succeeded in classifying 494 Samples accurately, while 2 samples were misclassified. For WU activity, the KNN classifier accurately classified all 471 samples with no misclassifications. IT correctly classified 413 samples out of the total number of 420 WD samples. However, the classification performance of the KNN is slightly degraded for ST and SD activities, where it has 469 accurately classified samples and 22 misclassifications for ST activity, and 499 accurate sample classifications out of the total number of 531 SD samples. Finally, the LD activity is accurately classified by the KNN classifier having only one wrong sample misclassified as ST activity. The preceding results of the KNN classifier lead to an overall classification accuracy of 97.82%.

As shown in the preceding results, the KNN classifier has high classification performance than the other classifiers, which is due to the following advantages:

- The KNN algorithm can compete with the most accurate models because it makes highly accurate predictions.
- Compared to other algorithms, it is very easy to predict multiclass problems. Supply the ‘k’ a value equivalent to the number of classes, and you are ready.
- It does not need to fit a model in advance; provide the data point, and it will give you the prediction.

Despite all these advantages, the KNN classifier is considered a lazy learning algorithm because it does not perform any learning mechanisms. It memorizes the training dataset instead, which leads to computational costs compared to other algorithms. However, it is still the better choice for applications where predictions are not requested frequently but where accuracy is important.

B. Discriminative Analysis (DA)classifier

**Figure 5:**
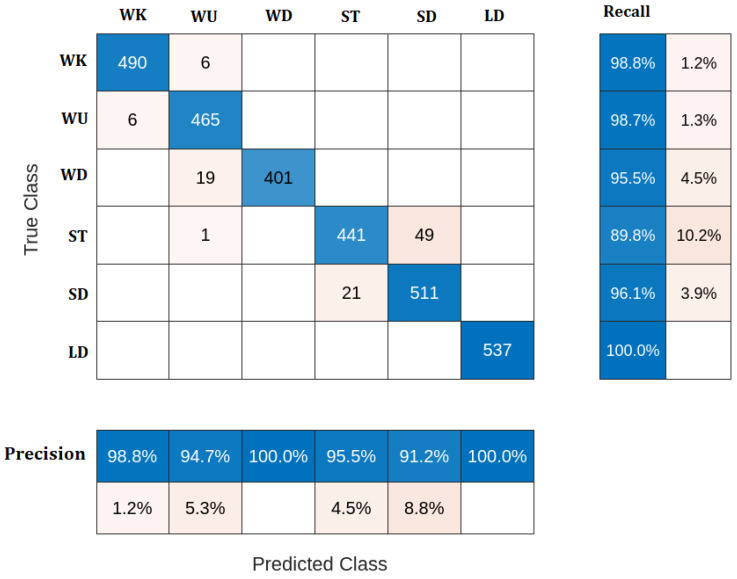
Confusion matrix of DA classifier.

Discriminant analysis (DA) is a classification technique that assumes that various classes produce data using Gaussian distributions. The trained classifier chooses the class with the lowest misclassification cost to determine the new data class. Figure 5 shows the confusion matrix of the DA classifier. The classifier successfully predicts the correct class for all the samples of LD activity. However, there are some misclassifications in the other activities. For example, for the WK activity, the classifier accurately classified 490 samples with 6 misclassifications. It accurately classified 465 WU samples out of the 471 samples. For the WD activity, 19 samples are misclassified as WU. The ST activity has the worst classification results, as the classifier misclassified 45 samples out of 491.

Additionally, the classifier classified 511 SD samples correctly, with 21 misclassifications. The obtained classification accuracy of the DA classifier is 96.53%. The DA classifier has advantages such as simplicity and low computational cost; however, it does not have the best classification performance compared to the other classifiers.

C. Naive Bayes (NB) classifier

**Figure 6:**
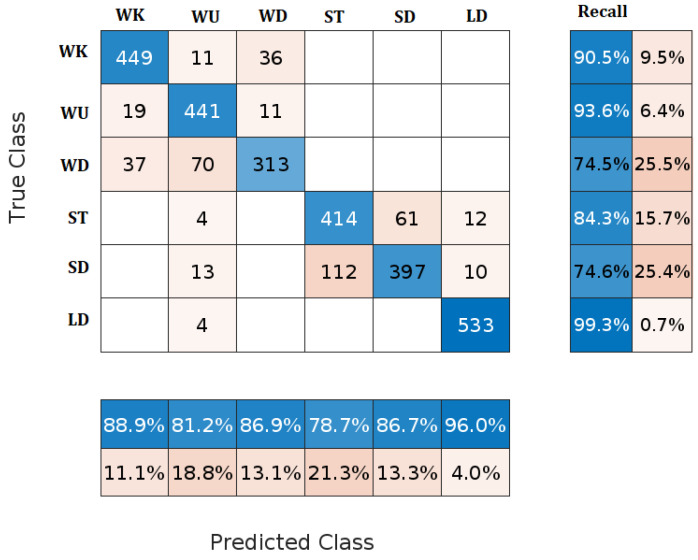
Confusion matrix of NB classifier.

The NB classifier is a probability-based classification method based on Bayes’ Theorem. It strongly assumes independence between features. The NB classifier determines the probability distribution of the target classes based on the features of the training set. It estimates the class of the new test data by determining the value closest to the observed probability. The obtained NB confusion matrix is shown in Figure 6. It can be seen in the figure that the NB classifier has degraded classification performance in comparison with KNN and DA classifiers because it has a large number of misclassifications. For example, the total number of samples in the SD activity is 532. The NB accurately classified only 397 samples and incorrectly classified 135 samples.

Additionally, many WD and ST samples are misclassified, decreasing classification accuracy. LD is the activity with the lowest number of misclassifications, where only 4 out of 537 LD samples are misclassified as WU. The overall classification accuracy of NB is 86.42%. The main advantage of the NB classifier is its low computational time needed for training; however, its classification accuracy is very low compared to other classifiers’ accuracy.

D. Decision Tree (DT) classifier

**Figure 7:**
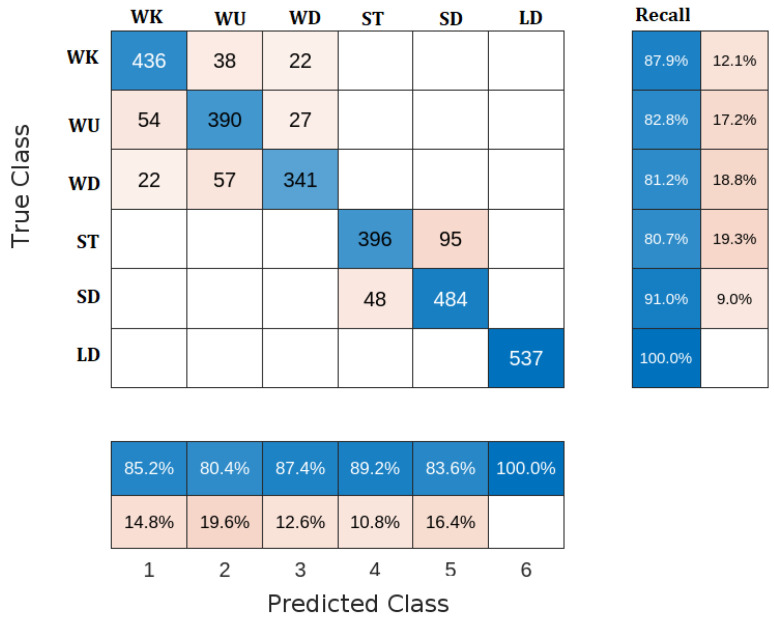
Confusion matrix of DT classifier.

DT is a classification technique that employs a decision tree to make predictions. The data are recursively partitioned into subsets according to the most significant feature at each tree node. It makes predictions using rules obtained from the features of the dataset. The confusion matrix of the DT classifier is shown in Figure 7. The DT confusion matrix shows that the DT classifier accurately classified all the samples of the LD activity. However, for other activities, it has several wrong classifications. For example, the WK activity accurately classified 436 samples, but the remaining samples were misclassified as 38 WU and 22 WD. For WU activity, it has 390 accurate classifications out of the total number of 471 samples. 341 WD samples are correctly classified out of 420 samples. Finally, the ST and SD activities have 95 and 48 misclassified samples. By comparing the results of DT with the previous classifiers, its classification performance is better than the NB classifier, but the KNN and DA classifier exceeds it. The accuracy of the DT classifier is 87.68%.

The advantages of DT classifier may include its simplicity and requiring little data preparation. However, it suffers from some limitations, such as requiring higher time to train the model and instability when small changes in the data occur.

**Figure 8:**
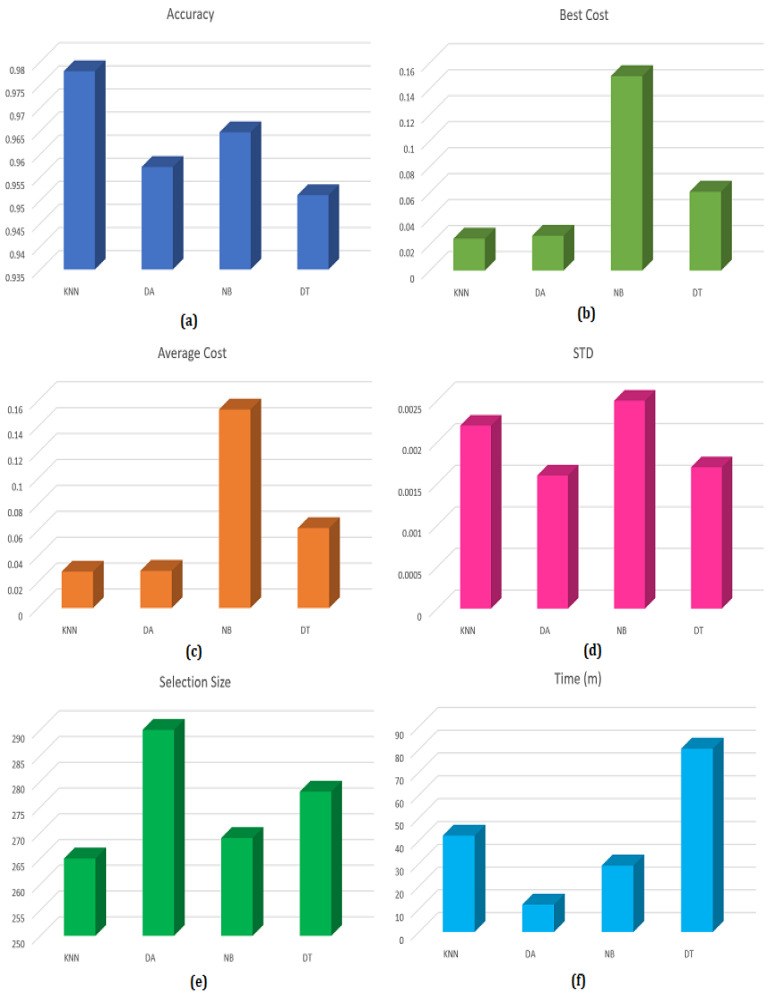
Comparison between different classifiers according to (**a**) accuracy, (**b**) best cost, (**c**) average cost, (**d**) STD, (**e**) selection size, and (**f**) time (min).

Because of the superior classification performance of the KNN classifier over the others, as seen in the previous analysis, we used the KNN classifier in the following experiments of our model. The recognition results of the proposed model are compared to eight existing FS techniques for the UCI-HAR dataset using the KNN classifier. The numerical results of the comparison are shown in Table 5. It is obvious from the comparison that the proposed approach achieved the best results for accuracy, best cost, average cost, and selection size with the values 0.9782, 0.024559, 0.0281, and 265, respectively. Although other algorithms, such as COA, AOA, SOA, and RSA, have better STD results than the proposed algorithm, they have more selection size and longer execution time. The bar charts in Figure 8 show a comparison between the four classifiers according to accuracy, best cost, average cost, STD, time, and selection size.

According to processing time, the proposed algorithm comes in the second order with a processing time of 42.2 min after RSA, with a processing time of 33.05 min. With a selection size of 265 out of 561 features, the proposed algorithm has proved its ability to achieve the highest reduction ratio of (52.7%).

**Figure 9:**
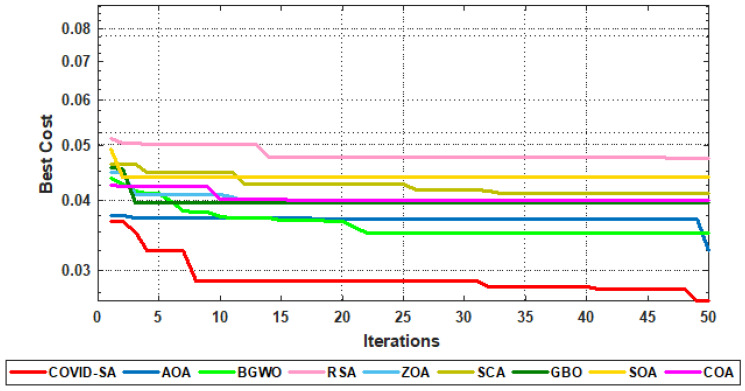
Convergence curves for all algorithms.

Figure 9 shows the convergence curves of the proposed and compared algorithms for HAR using the UCI-HAR dataset. The figure proves the superiority of the proposed algorithm as it has the minimum cost values.

The overall results prove the efficiency of the proposed model in the HAR process according to various metrics such as accuracy, precision, recall, best cost, average cost, STD, and execution time.

### 5.6. Comparison with Other Studies

For further evaluation of the proposed model, we compared the classification results of the proposed model with some recent HAR studies of the UCI-HAR dataset, such as [13,49,50]. In [13], the binary AOA algorithm is combined with CNN for optimal feature selection, and then the SVM is utilized to classify the selected features. This model achieved an average precision of 95.3%. The hybrid LSTM-CNN model proposed in [49] reported a mean precision of 95.8%. The LSTM model in [50] exceeds the two previous models with an average precision of 97.66%. The proposed model improved over these existing models by reporting an average precision of 97.9%. The proposed model achieved the highest precision for WK, WU, and LD, while all reported low precision for ST activity. Table 6 shows the results of the comparison.

## 6. Conclusions and Future Work

This work proposed an efficient HAR system based on data gathered from smartphones. A hybrid FS approach is developed to improve the performance of the HAR system. The proposed hybrid FS method combines the Simulated Annealing (SA) algorithm with the novel Coronavirus Disease Optimization algorithm (COVIDOA) to exploit their advantages and overcome limitations. Several classifiers are utilized to classify the features the proposed COVID-SA algorithm selects, and the KNN classifier shows superior performance. A comparison is conducted with several metaheuristics as FS methods using the KNN classifier. The proposed COVID-SA algorithm performed superior to other techniques according to various metrics such as classification accuracy, fitness value, STD, selection size, and processing time.

In future work, other classifiers, such as SVM and RF classifiers, may be used to classify human activities. Additionally, the proposed system can be applied to more complex HAR datasets.

## Figures and Tables

**Table 1:** Recent studies for HAR.

Ref.	Year	Dataset	Method	Average Accuracy
[11]	2020	UCI-HAR, WISDM	Deep learning with a hybrid ABCPSO algorithm	96.34% and 83.31%
[26]	2020	UCI-HAR	Hybrid feature selection model	96.81%
[31]	2020	UniMiB SHAR,	A semi-supervised deep learning framework	96%
[33]	2020	HAPT	CNN and LTSM	95.87%
[32]	2021	UCI-HAR	LSTM	93.519%
[9]	2021	UCI-HAR, WISDM	Hybrid gradient-based optimizer and grey wolf optimizer	98% and 98.87%
[15]	2022	N/A	Harris Hawks-optimized CNN	100%
[13]	2022	UCI-HAR, WISDM-HAR, and KU-HAR	Arithmetic optimization algorithm with deep learning	95.23%, 99.5%, and 96.8%
[38]	2022	WISDM, PAMAP2, and UCI-HAR	Ensemble measurement-based deep learning-based model	98.70%, 97.45%, and 95.05%
[39]	2023	UCI-HAR and WISDM	Feature selection and deep decision fusion	94.4% and 93.9%
[40]	2023	WISDM, UCI_HAR 2012, and PAMAP2	HDE and adaptive boosting with CNN	0.95%, 0.94%, and 0.95%

**Table 2:** Parameter setting for the state-of-the-art algorithms.

Algorithm	Parameter	Value
AOA	Exploitation parameter (α)	5
	Exploration paramter (µ)	0.5
GWO	Convergence parameter (a)	2 → 0
WOA	Convergence parameter (a)	2 → 0
	a2	−1 → −2
	B	1
RSA	Exploration accuracy for the hunting cooperation α	0.1
	Exploration accuracy for the encircling phase (β)	0.1
ZOA	Population movement parameter (I)	[1, 2]
GBO	Escape probability (pr)	0.5
SOA	Frequency control (fc)	0.1
	Correlation constant (µ)	0.001
	Correlation constant (v)	[0, 0.5]
	The angle (θ)	[0, 2π]

**Table 3:** Parameters for KNN, DT, and DA classifiers.

Classifier	Parameter	Description	Value
KNN	Distance	Distance function	[Euclidean, Manhattan, and Minkowski]
	K	Number of neighbors	[1, 2, 3, 4, …etc.]
DT	Criterion	Measure the quality of the data split.	[Gini, Entropy]
	Max Depth	[0, 2, 4, 6, 8]	The maximum depth of the tree.
DA	Gamma	Default: 0:0.1:1	control regularization parameter
	Delta	Default:0	control regularization parameter

**Table 4:** Comparison between different classifiers on the UCI-HAR dataset.

Classifier	Metric					
	Accuracy	Best Cost	AVG Cost	STD	Selection Size	Time (m)
KNN	0.9782	0.024559	0.0281	0.0022	265	42.2
DA	0.9653	0.026826	0.0287	0.0016	290	12
NB	0.8642%	0.14978	0.1533	0.0025	269	29.08
DT	0.8768	0.060721	0.0617	0.0017	278	80.31

**Table 5:** FS results of the proposed COVID-SA and the state-of-the-art algorithms.

Algorithm	Metric					
	Accuracy	Best Cost	AVG Cost	STD	Selection Size	Time (min)
Proposed COVID/SA	0.9782	0.024559	0.0281	0.0022	265	42.2
GWO	0.97048	0.034878	0.0364	0.0023	449	50.8
AOA	0.9722	0.0325	0.0369	6.3799 × 10^−4^	280	74.4
RSA	0.9661	0.047349	0.0483	0.0011	500	33.05
ZOA	0.966746	0.039590	0.0403	0.0017	533	53.5
GBO	0.9620	0.039601	0.0398	0.0012	476	80.2
SCA	0.9437	0.041117	0.0426	0.0025	320	45.3
SOA	0.96471	0.044014	0.0441	7.4229 × 10^−4^	415	83.5
COA	0.971496	0.040079	0.0405	8.9910 × 10^−4^	466	47.6667

**Table 6:** Comparison with state-of-the-art techniques.

Ref.	Method	Activity						Precision (%)
		WK	WU	WD	ST	SD	LD	
[13]	BAOA + CNN	99	97	99	89	88	100	95.3
[49]	LSTM + CNN	94.65	95.03	100	92.32	92.32	100	95.8
[50]	LSTM	99	96	99	95	99	98	97.66
Proposed	COVID-SA + KNN	99.2	99.2	99.8	93.4	95.8	100	97.9

## Data Availability

The dataset is available at: https://archive.ics.uci.edu/ (accessed on 10 May 2023).

## Abbreviations

List of abbreviations.

IoMT	Internet of Medical Things
IoT	Internet of Things
SHS	Smart Healthcare systems
HAR	Human Activity Recognition
FS	Feature selection
ABC	Artificial Bee Colony
WOA	Whale Optimization Algorithm
AOA	Arithmetic Optimization Algorithm
MPA	Marine Predators Algorithm
HHO	Harris Hawks Optimizer
COVIDOA	Coronavirus Disease Optimization Algorithm
SA	Simulated Annealing
STD	Standard Deviation
KNN	K-Nearest Neighbors
DA	Discriminant Analysis
NB	Naive Bayes
DT	Decision Tree
WK	Walking
WU	Walking upstairs
WD	Walking downstairs
ST	Sitting
SD	Standing
LD	Lying down
GWO	Grey Wolf Optimization
RSA	Reptile Search Algorithm
ZOA	Zebra Optimization Algorithm
GBO	Gradient-based Optimizer
SOA	Seagull Optimization Algorithm
SVM	Support Vector Machine
RF	Random Forest
CNN	Convolution Neural Network
TASKED	Self-KnowledgE Distillation
FDO	Fitness dependent optimizer
CSO	A competitive swarm optimizer for large-scale optimization
COA	Coyote Optimization Algorithm
SDBO	Supply–demand-based optimization
